# Effect of NAM-1 genes on the protein content in grain and productivity indices in common wheat lines with foreign genetic material introgressions in the conditions of Belarus

**DOI:** 10.18699/VJGB-23-26

**Published:** 2023-06

**Authors:** O.A. Orlovskaya, S.I. Vakula, K.K. Yatsevich, L.V. Khotyleva, A.V. Kilchevsky

**Affiliations:** Institute of Genetics and Cytology of the National Academy of Sciences of Belarus, Minsk, Belarus; Institute of Genetics and Cytology of the National Academy of Sciences of Belarus, Minsk, Belarus; Institute of Genetics and Cytology of the National Academy of Sciences of Belarus, Minsk, Belarus; Institute of Genetics and Cytology of the National Academy of Sciences of Belarus, Minsk, Belarus; Institute of Genetics and Cytology of the National Academy of Sciences of Belarus, Minsk, Belarus

**Keywords:** common wheat, wheat relatives, wheat introgressive lines, NAM-1 genes, grain protein content, productivity, мягкая пшеница, сородичи пшеницы, интрогрессивные линии пшеницы, гены NAM-1, содержание белка в зерне, продуктивность

## Abstract

Modern varieties of common wheat (Triticum aestivum L.) bred mainly for high productivity are often of low grain quality. The identification of NAM-1 alleles associated with high grain protein content in wheat relatives has enhanced the significance of distant hybridization for the nutritional value of T. aestivum L. grain. In this work we aimed to study the allelic polymorphism of the NAM-A1 and NAM-B1 genes in wheat introgression lines and their parental forms and evaluate the effects of various NAM-1 variants on the grain protein content and productivity traits in the field conditions of Belarus. We studied parental varieties of spring common wheat, the accessions of tetraploid and hexaploid species of the genus Triticum and 22 introgression lines obtained using them (2017–2021 vegetation periods). Full-length NAM-A1 nucleotide sequences of T. dicoccoides k-5199, T. dicoccum k-45926, T. kiharae, and T. spelta k-1731 accessions were established and registered with the international molecular database GenBank. Six combinations of NAM-A1/B1 alleles were identified in the accessions studied and their frequency of occurrence varied from 40 to 3 %. The cumulative contribution of NAM-A1 and NAM-B1 genes to the variability of economically important wheat traits ranged from 8–10 % (grain weight per plant and thousand kernel weight) to up to 72 % (grain protein content). For most of the traits studied, the proportion of variability determined by weather conditions was small (1.57–18.48 %). It was shown that, regardless of weather conditions, the presence of a functional NAM-B1 allele ensures a high level of grain protein content; at the same time, it does not significantly decrease thousand kernel weight. The genotypes combining the NAM- A1d haplotype and a functional NAM-B1 allele demonstrated high levels of productivity and grain protein content. The results obtained demonstrate the effective introgression of a functional NAM-В1 allele of related species increasing the nutritional value of common wheat.

## Abstract (ru)

Современные сорта мягкой пшеницы (Triticum aestivum L.), селекция которых велась в основном на увеличение продуктивности, часто имеют невысокое качество зерна. Обнаружение у сородичей пшеницы аллелей генов NAM-1, ассоциированных с высоким содержанием белка, увеличило значимость отдаленной гибридизации для повышения питательной ценности зерна T. aestivum L. Целью настоящей работы были изучение аллельного состава генов NAM-А1 и NAM-B1 у интрогрессивных линий пшеницы и их родительских форм и оценка эффекта различных вариантов генов NAM-1 на содержание белка в зерне и признаки продуктивности пшеницы в полевых условиях Беларуси. Исследовали родительские сорта яровой мягкой пшеницы, образцы тетраплоидных и гексаплоидных видов рода Triticum, а также 22 интрогрессивные линии, полученные с их участием (вегетационный период 2017–2021 гг.). Впервые установлены и зарегистрированы в международной молекулярной базе данных GenBank полноразмерные нуклеотидные последовательности гена NAM-A1 образцов T. dicoccoides к-5199, T. dicoccum к-45926, T. kiharаe, T. spelta к-1731. В исследуемой выборке выявлено шесть комбинаций аллелей NAM-A1/B1, частоты встречаемости которых варьировали от 40 до 3 %. Совместный вклад генов NAM-A1 и NAM-B1 в изменчивость хозяйственно важных признаков пшеницы составил от 8–10 % (масса зерна с растения и мас- са 1000 зерен) до 72 % (накопление белка в зерне). Для большинства изученных признаков доля изменчивости, обусловленная погодными условиями, была невелика (1.57–18.48 %). Показано, что наличие функционального аллеля NAM-В1 обеспечивает высокий уровень накопления белка в зерне независимо от погодных условий и при этом не приводит к существенному снижению массы 1000 зерен. Высокие показатели продуктивности и уровня накопления белка в зерне установлены для генотипов, сочетающих гаплотип NAM-А1d и функциональный аллель NAM-B1. Полученные результаты свидетельствуют об эффективности интрогрессии функционального аллеля гена NAM-В1 от видов-сородичей для повышения питательной ценности мягкой пшеницы.

## Introduction

Common wheat (Triticum aestivum L.) is an important agricultural crop that plays a key role in providing food to people across the globe. One of the priority directions of wheat breeding is to improve grain quality, which is primarily determined by the total protein content (Brevis et al., 2010). Complex polygenic nature of the trait “grain protein content”, its variability when exposed to external factors, as well as the negative correlation between the protein content and productivity, complicate the breeding process (Iqbal et al., 2016). In addition, low genetic diversity of modern varieties by trait limits their use in breeding programs aimed at improving the nutritional value of wheat. Many related species of T. aestivum are characterized by a higher grain protein content compared to cultivated varieties (Peleg et al., 2008; Kumar et al., 2019).

New opportunities that make it possible to increase the total grain protein appeared in breeding with the identification of the Gpc-B1 locus associated with protein content in wild emmer T. dicoccoides (AABB genome). The locus was mapped in the short arm of chromosome 6B and upon detailed clarification of its localization site boundaries, a sequence was found identified as the NAM-B1 gene belonging to NAC family transcription factors (Uauy et al., 2006a). The genes of this family are involved in the regulation of various plant development programs, control of defense responses to biotic and abiotic stressors, and they play an important role in plant senescence (Puranik et al., 2012; Zhao et al., 2015). In addition to the NAM-B1 gene, common wheat also has homologous NAM-A1 and NAM-D1 genes on chromosomes 6A and 6D (Avni et al., 2014).

A functional NAM-B1 allele (wild-type allele) was found in wild emmer, providing high protein content in grain. The allele includes three exons and two introns and encodes a protein of 407 amino acid residues that has the conserved N-terminal region, or the NAC domain with five subdomains and the highly variable C-terminal transcriptional activation region (Waters et al., 2009). A functional NAM-B1 allele is not found in most modern wheat varieties. Varieties have, as a rule, a 1 bp insertion in the first exon leading to a frameshift (mutant allele) or a gene deletion (partial or complete) and, as a result, to an inactive protein or its absence (Uauy et al., 2006b). Thus, a study of 218 wheat varieties from five main regions of China did not reveal any single variety with a functional allele of the NAM-B1 gene (Chen et al., 2017). Molecular characterization of the NAM-1 genes of Australian common wheat varieties showed the presence of the wild allele of the NAM-B1 gene in only 2 out of 51 varieties (Yang et al., 2018).

It was established that the NAM-A1 gene similar to NAM- B1 consists of three exons; it possesses typical characteristics of NAC-family genes and is involved in the regulation of the same processes as NAM-B1. As a result of the analysis of the association of single nucleotide variants (SNPs) of the NAM-A1 gene with nitrogen remobilization from leaves and grain protein accumulation, two functional single nucleotide substitutions were identified: at position 722 (T/C) and at position 1509 (A/del). Based on the data obtained, a classification of NAM-A1 haplotypes was proposed: NAM-A1a (722C and 1509A), NAM-A1b (722C and 1509del), NAM-A1c (722T and 1509A), and NAM-A1d (722T and 1509del) (Cormier et al., 2015).

The absence, in modern wheat varieties, of the functional NAM-B1 allele, which provides for high grain protein content in various environmental conditions, has strengthened the position of distant hybridization from a perspective of increased nutritional value of wheat grain. In order to enrich and to improve the common wheat gene pool, in the crossing with T. aestivum L. varieties we used accessions of the species of the genus Triticum (T. dicoccoides, T. dicoccum, T. durum, T. spelta, and T. kiharae). An earlier study of genetic diversity of the collection of introgressive wheat lines using C-banding and SSR analysis showed that in the genome of hybrid lines the foreign genetic material is presented both in the form of short fragments and whole chromosomes (Orlovskaya et al., 2016, 2020).

In this work, we aimed to study the allelic composition of NAM-A1 and NAM-B1 genes in introgressive wheat lines and their parental forms and to evaluate the effect of different variants of NAM-1 genes on grain protein content and wheat productivity traits in the field conditions of Belarus.

## Materials and methods

The study included five varieties of spring common wheat (Rassvet, Saratovskaya 29, Festivalnaya, Belorusskaya 80, and Pitic S62); tetraploid accessions T. dicoccoides, T. dicoccoides k-5199, T. dicoccum k-45926, and T. durum, and hexaploid accessions T. spelta k-1731 and T. kiharae of the species of the genus Triticum, as well as 22 introgressive lines we had developed (Supplementary Material 1)1. The accessions of foreign donors were obtained from the collection of N.I. Vavilov All-Russian Institute of Plant Genetic Resources (VIR). Information about the pedigree of individual accessions is not available as unpreserved (VIR catalogue numbers are not indicated).

Supplementary Materials are available in the online version of the paper: http://vavilov.elpub.ru/jour/manager/files/Suppl_Orlovskaya_Engl_27_3.pdf.


The plants were grown in the experimental fields of the Institute of Genetics and Cytology of the National Academy of Sciences of Belarus, in 2017–2021 (Minsk), on sod-podzolic loamy sand soil. The characteristic of weather conditions in the region of our experiment in 2017–2021 is presented in Supplementary Material 2. Data on average daily temperatures and precipitation (http://rp5.by) were used to calculate the sum of active temperatures (SAT) and Selyaninov’s hydrothermal coefficient (HTC) (Mamontova, Khromov, 1974). When harvesting, the following traits were taken into account: plant height, the number of productive shoots per plant, the length of the main spike, the number of spikelets and grains of the main spike, grain weight per spike and plant, as well as thousand kernel weight. To assess the traits, 15 plants of each genotype were randomly selected.

To sequence full-length NAM-A1 gene sequences and the first exon of NAM-B1, specific primers developed by R. Yang et al. (2018), were used. The sequencing reaction was performed using the BigDye Terminator v. 3.1 Cycle Sequencing kit (Applied Biosystems, USA); the separation of sequencing reaction products was carried out using the ABI PRISM 3500 genetic analyzer (Applied Biosystems). Alignment of nucleotide sequences and homology analysis were performed using the BLAST analyzer of the National Center for Biotechnology Information, USA (http://www.ncbi. nlm.nih.gov/BLAST). The Chinese Spring variety was used as a reference sequence, which, according to the literature data, is the carrier of the NAM-A1a haplotype (722C and 1509A) and the NAM-B1 mutant allele (T insertion at position +11) (Yang et al., 2018).

The total protein content in wheat grain was determined in accordance with GOST 10846-91 (2009) at the Central Republican Laboratory of the State Institution “State Inspectorate for Testing and Protection of Plant Varieties” (Minsk, Belarus). The essence of the method lies in the mineralization of organic matter with sulfuric acid in the presence of a catalyst with the formation of ammonium sulfate, the destruction of ammonium sulfate with alkali with the release of ammonia, the stripping of ammonia with water vapor into a solution of sulfuric or boric acids followed by titration.

The results of the experiment were summarized using descriptive statistics methods; two-way analysis of variance, regression, and correlation analyses (the Spearman’s rank correlation coefficient was used). Statistical procedures were implemented in the software packages Statistica 10.0, MS Excel, and web application SNPstats. A quantitative contribution of individual factors of the dispersion analysis was calculated on the basis of the relation of the absolute factor variance to the sum of variances of that factor and other factors, according to the formulae given in (Rokitsky, 1973).

## Results

Allelic polymorphism of NAM-A1 and NAM-В1 genes

Full-length NAM-A1 gene sequencing was carried out in the parental varieties and accessions of species of the genus Triticum, in 22 introgressive lines developed on their basis. Among the parental forms, we detected haplotype NAM-A1a in Festivalnaya and Rassvet varieties; accessions T. durum, T. dicoccum k-45926, T. dicoccoides k-5199, T. dicoccoides, and T. kiharae; haplotype NAM-A1c in T. spelta k-1731; and haplotype NAM-A1d in varieties Saratovskaya 29, Belorusskaya 80, and Pitic S62 (Supplementary Material 3).

A comparative analysis showed that NAM-A1a nucleotide sequences of T. dicoccoides k-5199, T. dicoccoides, and T. kiharae accessions did not have 100 % similarity with the NAM-A1a sequence of T. aestivum (MH160778) from the GenBank database. The sequences of wild emmer accessions (T. dicoccoides k-5199 and T. dicoccoides) that we studied differed from NAM-A1а of T. aestivum (MH160778) in two SNPs: positions 538 bp (C/A in exon 2) and 1139 bp (G/T in exon 3) (99.9 % identity level). The SNP 1139 bp of G/T results in the replacement of asparagine with tyrosine in the amino acid sequence of protein. The level of similarity of NAM-A1а of the T. kiharae accession with NAM-A1а from the GenBank database (MH160778) was 99.7 % and differed from it in six SNPs: in the positions of 189 bp (C/A in exon 1), 306 bp (A/C in intron 1), 1133 bp (G/A in exon 3), 1271 bp (G/T in exon 3), 1414 bp (C/G in exon 3), and 1491 bp (G/C in exon 3). Three of these SNPs lead to changes in the amino acid sequence of the protein: a G/A substitution in the position of 1133 bp leads to the replacement of alanine with threonine; G/T in the position of 1271 bp results in the replacement of alanine with serine; and G/C in the position of 1491 bp replaces glycine with alanine.

The NAM-A1 gene sequence of T. dicoccum k-45926 and T. durum accessions was completely homologous to the NAM- A1a allele sequence (GenBank: MH160778); the NAM- A1 sequence of the T. spelta k-1731 accession corresponded to the NAM-A1c allele (GenBank: MH MH160777). Nucleotide sequences of the NAM-A1 gene of T. kiharae, T. spelta k-1731, T. dicoccoides k-5199, and T. dicoccum k-45926 accessions, which we described for the first time, were registered with the International GenBank Database (access codes MT572492, MT920417, MW384855, and MW384856, respectively).

Analysis of NAM-A1 gene sequencing data in introgressive wheat lines showed that 54.6 % of the lines had the NAM-A1d haplotype; 36.4 % – the NAM-A1a haplotype; and 9.1 % – the NAM-A1c haplotype. It should be noted that wheat lines with foreign genetic material inherited, as a rule, the NAM-A1 gene of the original wheat variety but there were a number of exceptions (see Supplementary Material 3). A haplotype corresponding to a related species was identified in the lines developed using T. spelta k-1731 (lines 1-8 and 7, NAM-A1c); in line 226-7 T. durum × Belorusskaya 80 (NAM-A1a); and in line 20-1 T. kiharae × Saratovskaya 29 (NAM-A1a). Among the lines developed using T. dicoccoides, only lines 11-1 and 13-3 obtained as a result of crossing with the Festivalnaya variety inherited the NAM-A1 gene from wild emmer with the SNPs only characteristic of it at positions 538 bp and 1139 bp of the nucleotide gene sequence.

Analysis of the sequenograms of the first exon of the NAM-B1 gene in the studied genotypes revealed a functional allele (F ) in all the accessions of related species, except for T. durum, for which the NAM-B1 allele was not identified, and in 5 out of 22 introgressive wheat lines (13-3 and 15-7-1 of the T. dicoccoides × Festivalnaya combination; 19 and 25-2 of T. kiharae × Saratovskaya 29; and line 7 of the T. spelta k-1731 × Saratovskaya 29 combination). All parental varieties and most of the wheat lines with the foreign genetic material (77.3 %) had a mutant allele (NF ).

Effects of NAM-A1 and NAM-B1 genes

on the productivity traits and the grain protein content Genotyping results of common wheat introgression lines and parental forms by NAM-A1 and NAM-B1 genes were compared with the results of field trials and the data on grain protein content for 2017–2021. The effect of genotype, environmental factors, and their interaction were assessed using the general linear model (GLM) of two-way analysis of variance (Supplementary Material 4).

The combination of NAM-A1/B1 genes produces a statistically significant effect on the manifestation of all nine traits studied, while exceeding a contribution of individual NAM-1 genes to the variability of protein content, plant height, the number of spikelets per spike, and thousand kernel weight (see Supplementary Materials 4 and 5). NAM-B1 does not significantly affect the spike length of wheat, while NAM-A1 or the combination of NAM-A1/B1 determine more than half of the observed trait variation

The greatest length of the main spike relative to other haplotypes is typical for NAM-A1c plants (9.86 cm in average over the 5-year observation period), and in the case of the c/F combination, this indicator increases up to 10.87 cm. The effect of NAM-B1 allelic variants on the variability of the number and weight of grains in the spike is almost three times higher than the effect of NAM-A1 haplotypes, and it is one-and-a-half times higher in the case of the NAM-A1 and NAM-B1combination

The spike productivity in the group of samples with the wild NAM-B1 allele was significantly lower than in the genotypes with a mutant allele (Supplementary Material 6). Thus, in the spike of the vast majority of genotypes with a functional allele (7 out of 10), the number of grains did not reach 30 pieces, while in the spike of genotypes with a mutant allele, as a rule, 30–40 grains were formed. A significant variation in spike productivity traits can be noted in both groups. Individual genotypes with a functional allele demonstrated high indices by these traits (lines 19 of T. kiharae × Saratovskaya 29 (d/F ) and 15-7-1 of T. dicoccoides × Festivalnaya (a/F )). In all three variants of variance analysis, thousand kernel weight and the weight of grains per plant are the traits with a low (up to 10 %) contribution of genetic variances; at that, the weight of grains per plant is statistically independent of the NAM-A1 haplotype and thousand kernel weight is independent of the NAM-B1 allele. Variability of the protein content in grain is 70 % associated with NAM-B1 polymorphism, and a contribution of NAM-A1 is significantly lower and it slightly increases when considering a combination of gene alleles (see Supplementary Material 5). Thus, on average, during the 4-year period, the highest grain protein content in the groups with different NAM-A1 haplotypes was observed in the case of NAM-A1a genotypes (21.53 %); in the groups with different NAM-B1 variants – in the case of the functional NAM-B1 allele (22.53 %); and the maximum amount of protein was noted (23.72 %) in the case of the a/F combination (Supplementary Material 7).

The strength and direction of the relationship between the different alleles of NAM-1 genes and economically valuable traits was assessed using the Spearman’s correlation coefficient (Table 1).

**Table 1.**
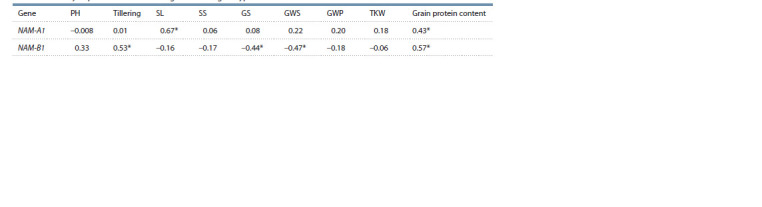
Spearman’s correlation coefficient among the allelic NAM-А1 and NAM-В1 gene variants and economically important traits of investigated wheat genotypes

A medium-strength relationship was found between the grain protein content and the allelic variants of NAM-1 genes, but only the correlation with NAM-B1 is statistically significant (see Table 1). Apart from that, significant dependence was established between the NAM-B1 allelic variants and the tilling capacity, the number and weight of grains per spike. The NAM-A1 haplotype significantly correlated only with the spike length; association with other productivity traits was weak. Variance analysis results also showed that NAM-B1 allelic variants produce a significantly greater impact on the variability of grain protein content, the number and weight of grains per spike, and productive tilling capacity than NAM-A1 (see Supplementary Material 5). It should be noted that both variance and correlation analyses established a low correlation of NAM-1 genes with the traits “plant height”, “the number of spikelets per spike”, “grain weight per plant” and “thousand kernel weight” (see Supplementary Material 5, Table 1).

In total, six combinations of NAM-A1/B1 alleles were identified in the accessions under study and their frequencies varied from 40 (d/NF) to 3 % (c/NF ). Some combinations of alleles are represented by a small number of samples (d/F, c/F, and c/NF ), and therefore, it does not seem possible to speak about a significant difference of traits in plants with such combinations of alleles

Mean values of productivity traits of the genotypes carrying various combinations of NAM-A1/B1 genes are demonstrated in Table 2.

**Table 2.**
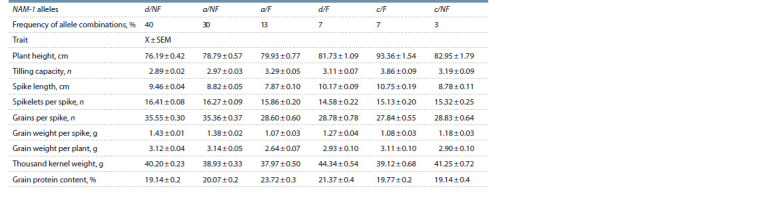
Mean values of productivity traits and grain protein content in the groups of wheat genotypes with different combinations of NAM-1 allelic variants Notе. а – NAM-A1a haplotype; d – NAM-A1d haplotype; c – NAM-A1c haplotype; F – functional NAM-B1 allele; NF – mutant NAM-B1 allele; X – mean values of traits; SEM – the standard error of the mean.

The genotypes combining different NAM-A1 haplotypes with the functional NAM-B1 allele were taller and of a higher tilling capacity than the plants with a corresponding haplotype combined with the mutant NAM-B1 allele (see Table 2). The maximum values for these traits are typical for c/F accessions: 93.36 cm and 3.86 pcs. An opposite trend was revealed for spike productivity traits: all three NAM-A1 haplotypes in combination with the functional NAM-B1 allele had a low number and weight of grains per spike (see Table 2). The samples with NAM-A1a and NAM-A1c haplotypes in combination with the functional allele demonstrated a slight decrease in thousand kernel weight compared with the genotypes combining NAM- A1a and NAM-A1c and the mutant allele (see Table 2).

For the plants with the NAM-A1d haplotype, an increase in thousand kernel weight in the group with the functional allele was revealed (40.20 g for d/NF genotypes and 44.34 g for d/F genotypes). It can be noted that NAM-A1a accessions were the least productive by this trait both in the group with functional (37.97 g) and non-functional (38.93 g) NAM-B1 alleles. The presence of the mutant allele of the NAM-B1 gene leads to a decrease in the protein content relative to the combination with the functional allele: for NAM-A1а haplotypes – by 3.6 % on average; for NAM-A1d by 2.3 %; and for NAM-A1c, by 0.6 % (in the latter case, the decrease is not statistically significant). The maximum amount of protein in grain was accumulated by the lines with the a/F combination and the minimal amount – by the lines with the d/NF and c/NF combinations (see Table 2).

The role of weather conditions and their relationship with NAM-1 genes regarding variability of productivity traits and grain protein content

Regardless of the model used (NAM-A1 × weather conditions, NAM-B1 × weather conditions; and NAM-A1/B1 × weather conditions), the high statistical significance of the contribution of weather conditions of the year of growth was shown for all the traits. However, the predominant role of this factor (> 50.0 %) was found only in the variability of traits “plant height” and “the number of spikelets per spike”. The interaction of the NAM-A1/B1 gene combination with weather conditions significantly affects all wheat productivity indices. The contribution of this interaction is especially high (more than 60 %) in the variance of the traits “grain weight per spike and plant”, “thousand kernel weight” and “tilling capacity”. The impact of the interaction “NAM-B1 × weather conditions” on productivity traits is lower than the contribution of other factors (see Supplementary Material 5). The only exception is the trait “number of spikelets per spike”, for which a contribution of genotype-environmental interactions with NAM-B1 is 3 % and with NAM-A1 it is statistically insignificant ( p = 0.23). It should be noted that the interaction of weather conditions and all three genetic factors did not affect grain protein content.

Different genotypes may respond to changes in environmental conditions differently. Additivity of NAM-A1/B1 effects and weather conditions on the manifestation of the traits studied was tested using the log-additive linear regression model of the SNPstats web application. For the traits “plant height”, “tilling capacity”, “the number of spikelets per spike”, “weight of grains per plant”, and “thousand kernel weight”, the mutual enhancement of the effects of two factors was found: NAMA1/ B1 alleles and weather conditions. The additive interaction of the NAM-A1/B1 genotype and the environment is statistically insignificant for the variability of traits “spike length”, “the number and weight of grains in the main spike”, and “protein content”.

Variability of the studied traits of genotypes carrying various NAM-A1/B1 combinations was assessed under the conditions of different growing seasons relative to the productivity of plants with d/NF alleles in 2017 (see the Figure). With a view to searching for meteorological factors that determine genotype-environmental interaction, the following was carried out: (1) a comparative analysis of the ranking of allelic combinations in 2017–2021 conditions; (2) a correlation analysis of productivity traits with meteorological parameters (see the Figure).

**Fig. 1.**
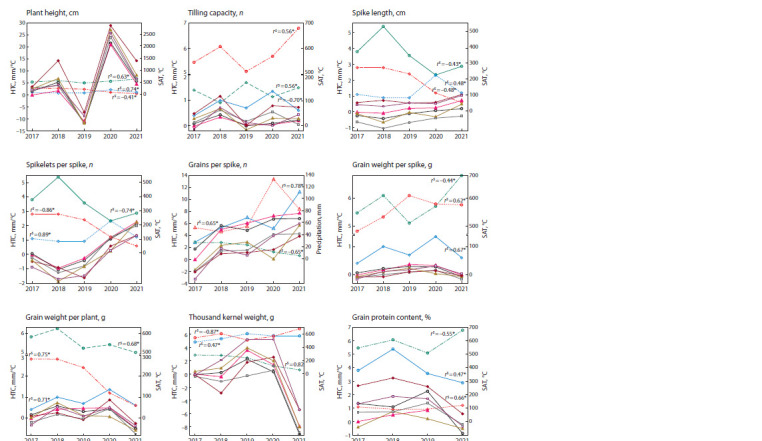
The difference in productivity traits between the wheat plants with d/NF in 2017 and different combinations of NAM-A1/B1 alleles under the weather conditions of Belarus in 2017–2021 (SNPStats results). Combinations of NAM-A1/B1 alleles: d/NF; a/NF; a/F; c/F; d/F; c/NF. Weather conditions: EHS – effective heat sum; HTC – hydrothermal coefficient; – rainfall. Vegetation periods: May; June; July; August; r s – the Spearman’s rank correlation coefficient, which shows the strength of the relationship between productivity traits and meteorological parameters.

An analysis of correlation coefficients showed that most of the traits studied were significantly influenced by weather conditions during the grain filling stage – HTC and SAT in July. The closest association was found between the HTC in July and the plant height, the number of spikelets per spike, the weight of grains per plant and thousand kernel weight (see the Figure). During the grain filling stage, there is an increased supply of mineral and organic substances to the wheat grain; unfavorable conditions during this period significantly worsen its quality and reduce yields. The optimal HTC in July (1.2) is typical for the 2020 season. In July 2021, there was an increase in air temperature and a lack of precipitation compared to the norm, and the HTC was only 0.4, characterizing this period as dry. In other years, the HTC in July significantly exceeded the norm and amounted to 2.8, 2.8 and 2.4 in 2017, 2018 and 2019 respectively. It can be noted that the air temperature and precipitation throughout the growing season of 2020 were close to the climatic norm, which contributed to the maximum realization of wheat productivity (Supplementary Material 8). The conditions of 2021 (soil waterlogging in May; drought in July; and rains during grain ripening and harvesting) led to a significant decrease in the yields of the genotypes under study (see Supplementary Material 8).

“Plant height” is a trait with an additive contribution of NAM-1 genes and weather conditions. The presence of the functional NAM-B1 allele and the NAM-A1c haplotype is associated with a statistically significant increase in culm. Maintained soil moisture (HTC > 1) during the “booting– flowering” period stimulates plant growth, while excessive precipitation in July-August, on the contrary, negatively affects plant height. For example, the average height of wheat plants in 2019 (cold and wet summer) decreased by 10.6–14.5 cm, and in 2020 (the year close to the climatic norm) it increased by more than 20 cm relative to 2017 values. Plants with the NAM-A1c haplotype are the least sensitive to environmental factors that reduce the stem height, while the growth of the culm reaches 10–25 cm under favorable conditions.

It was shown that the tilling capacity of plants is negatively affected by soil waterlogging and a large amount of precipitation in August, which is associated with the inhibition of secondary growth processes, lodging and disruption of gas exchange in the root system. The trait is positively correlated with the HTC of May and the SAT of July; that is, the absence of frosts and drought at the tillering stage and the high intensity of photosynthesis at the maturation stage. Plants with the functional NAM-B1 allele form more stems under favorable conditions and significantly reduce tillering capacity under spring drought conditions (see the Figure).

SNPstats models failed to explain spike length variability either by the additive interaction of NAM-A1 and NAM-B1 alleles or the interaction of genetic factors and growing conditions. Both the spike length and the number of spikelets per spike are influenced by the conditions observed at the stages of booting and heading, namely, the HTC in June. Warm and dry conditions in May-June 2018 were accompanied by a decrease in the average length and the number of spikelets per spike. However, for late-ripening genotypes with the c/F combination, a decrease in the number of spikelets turned out to be statistically insignificant. The number and weight of grains in the main spike depend on the optimal moisture conditions at the stage of germination and tillering, the sum of active temperatures at the stage of heading and flowering. Low yields of the main spike were noted in the conditions of 2017 and 2021, which were characterized by a low hydrothermal coefficient in May, but differed in temperature and hydrological regimes at the subsequent stages of the growing season. In 2021, the maximum number of grains formed in the main spike was noted and in 2017 – the minimum one (see the Figure).

The weight of grains per plant and thousand kernel weight are the most important indicators of wheat yield. In cases of a significant difference in the mean values of traits in the plants carrying NAM-A1/B1 combination variants, genetic factors do not produce an additive effect. Regardless of the year of cultivation, NAM-A1a and NAM-A1c haplotype combinations with a functional NAM-B1 variant are associated with a slight decrease in thousand kernel weight relative to combinations with a non-functional gene variant. There were no significant differences found between d/F and d/NF genotypes by seed productivity traits (see the Figure). Late-ripening genotypes carrying the NAM-A1с haplotype significantly increase thousand kernel weight in the case of a combination of high HTC in July and August, which was observed in 2019–2020 (see the Figure).

For the formation of grain, wheat plants remobilize nitrogen and carbohydrates from the flag leaf, and therefore, the protein content in grain depends on the intensity of photosynthesis and the photosynthetic surface area (Lawlor et al., 1989). High HTC in June may produce a negative effect on the protein content through a decrease in these parameters – low solar insolation as a result of high cloudiness and an increase in the lamina damage area caused by phytopathogens under high humidity conditions. Under unfavorable conditions for overall productivity in 2021, plants with the functional NAM- B1 allele accumulated more protein than the genotypes with a mutant gene variant (see the Figure).

## Discussion

The common wheat varieties we studied had the NAM-A1d or NAM-A1a haplotype and the mutant allele NAM-B1, which is consistent with the literature data. For example, in the collection of Australian wheat varieties, accessions with haplotypes NAM-A1a (50.1 %) and NAM-A1d (28 %) were the most frequently occurring, while NAM-A1b (1.9 %) was the least common (Yang et al., 2018). F. Cormier et al. (2015) revealed in their studies that the NAM-A1d haplotype is typical for most modern European wheat varieties, while NAMA1a is more common among the varieties with high baking properties. Wheat samples with the NAM-A1b haplotype were not found in the collection we studied. The works of foreign scientists provide data on the low frequency of occurrence of this haplotype. For example, in the collection of 795 wheat accessions, only one accession with NAM-A1b was found. There is an assumption that this haplotype has appeared recently as a result of recombination between NAM-A1a and NAM-A1d (Cormier et al., 2015).

The presence of functional NAM-B1 alleles only in wheat relatives among the parental forms we studied is confirmed by the data of other researchers. Thus, in the work of C. Uauy et al. (2006b), a functional NAM-B1 allele was found in all 42 analyzed accessions of T. dicoccoides and in 17 out of 19 accessions of T. dicoccum (Schrank) Schuebl., while all 57 studied varieties of durum wheat and 34 of common wheat either contained a 1 bp insertion or had a gene deletion. Among 367 common wheat accessions of the INRA core collection (France) selected from 3942 genotypes of different geographic origin, only 5 contained functional NAM-B1 alleles (Hagenblad et al., 2012). Due to the fact that currently cultivated varieties are, as a rule, missing a functional NAM-B1 allele, five introgressive lines of wheat with the allele that we have developed are of great interest from the point of view of their ability to enhance the quality of wheat grain.

Analysis of differences in the mean values of quantitative traits in the groups carrying different NAM-A1 haplotypes showed that during all years of the experiment, NAM-A1a plants had a short spike, low grain weight per plant and thousand kernel weight, and high protein content in grain; NAM-A1c – the maximum plant height, tilling capacity and spike length but low grain content and seed weight per spike; NAM-A1d – the minimum plant height, number of productive shoots, and the maximum values of spike and plant productivity traits. A number of studies have shown that the presence of the NAM-A1a haplotype is associated with a shorter period of grain filling and a more intense process of nitrogen remobilization, which leads to increased protein content; but, at the same time, to a decrease in the number of grains per spike and thousand kernel weight, while the presence of NAM-A1c or NAM-A1d leads to an increase in the grain filling period, which results in an increase in the amount of nitrogen uptake and wheat yield (Cormier et al., 2015; Alhabbar et al., 2018). There is an assumption that NAM-A1a is a functional variant of the NAM-A1 gene, which is rarely found in elite modern wheat varieties the breeding of which was carried out mainly for productivity purposes

The data we obtained on a high level of protein accumulation in grain in wheat genotypes with the wild NAM-B1 allele coincide with the results of many foreign scientists. The study of a series of almost isogenic lines based on common and durum wheat in different countries across the globe (the USA, Argentina, India, China, Australia, etc.) allowed us to conclude that the introgression of a functional NAM-B1 allele into the genome of cultivated wheats of both ploidy levels leads to an increase in the content of protein and key minerals in grain, an improved harvesting nitrogen index, and increased protein harvest (Tabbita et al., 2013; Maphosa et al., 2014; Mishra et al., 2015; Kuhn et al., 2016).

Throughout the entire observation period, the genotypes with a functional NAM-B1 allele were characterized by higher plant height and tilling capacity, but lower indicators by spike and plant productivity traits compared to the genotypes carrying a nonfunctional allele. However, we did not find any significant impact of the NAM-B1 allele state on thousand kernel weight. In the literature, there are data on both positive and negative effects of the wild-type NAM-B1 allele on the main components of wheat productivity (Carter et al., 2012; Maphosa et al., 2014; Kuhn et al., 2016). A significant increase in the productive stems in common wheat lines with a functional NAM-B1 allele that we established was also described in the works of other scientists (Tabbita et al., 2013; Vishwakarma et al., 2016).

Tillering capacity is known to be determined by many environmental factors, including nitrogen availability (Wang, Below, 1996). It is possible that a functional NAM-B1 allele contributes to the formation of productive stems due to the fact that it improves nitrogen metabolism (Tabbita et al., 2013). According to the review article that summarizes the data of 25 studies on the influence of allelic variants of the NAM-B1 gene on 50 wheat traits, 36 % of the studies did not show any significant differences in thousand kernel weight between the genotypes with different allele variants of this gene; correspondingly, 23 and 41 % of the studies revealed both a significant decrease and a significant increase in this indicator in the lines with a functional allele. It should be noted that the majority of studies (79 %) did not establish a statistically significant effect of NAM-B1 polymorphism on wheat yield, and only 4 % showed a decrease in the yield of lines with a functional allele (Tabbita et al., 2017). This fact is explained by a positive effect of a functional allele on the formation of productive stems, since it is precisely due to high tillering capacity that no significant decrease takes place in grain yield, even despite low spike productivity (Tabbita et al., 2013).

Evaluation of the effect of six combinations of NAM-A1/B1 alleles on the level of manifestation of a number of economically valuable wheat traits showed that the maximum height and tillering capacity are characteristic of c/F genotypes; d/ NF genotypes are responsible for high productivity of spikes and plants; and the highest grain protein concentration is determined by a/F (see Table 2). Similar results are presented in (Alhabbar et al., 2018), regarding the influence of the allelic composition of NAM-1 genes on the efficiency of nitrogen use, productivity and protein content in wheat grain. In this study, the Mace variety, which has a non-functional NAM-B1 allele and the NAM-A1d haplotype, significantly outperformed other genotypes in terms of yield but had the minimum grain protein content. Varieties combining a functional allele with different NAM-A1 haplotypes had high grain protein content, while a negative correlation was found between the mutant allele and grain protein content, regardless of the NAM-A1 haplotype (Alhabbar et al., 2018).

The combination of NAM-A1/B1 genes had a significant effect on the formation of all the traits of wheat under study, while the grain weight per plant was statistically independent of the NAM-A1 haplotype, and the spike length and thousand kernel weight were statistically independent of the NAM-B1 gene allele. It is also important to note a small joint contribution of these two genetic factors to the variability of most productivity traits studied (7.59–18.75 %), but the predominance of NAM-A1/B1 in the variability of protein content in grain (72 %) should be mentioned at that.

It is known that, in addition to the genetic control, crop and its components are significantly influenced by environmental factors (Ahmed et al., 2020; Kronenberg et al., 2021). Our study shows a high statistical significance of the contribution of weather conditions to the variability of wheat quantitative traits. A particularly high role of this factor (more than 50 %) was revealed for the dispersion of traits with a wide reaction rate and a high variation coefficient strongly dependent on the ambient temperature and the amount of precipitation – “plant height” and “the number of spikelets per spike”. For the rest of the traits studied, the share of variability determined by weather conditions was significantly lower and was in the range of 1.57–18.48 %; and the impact of the “NAM-A1/ B1 × weather conditions” interaction was of great importance (> 60 %). According to the data we obtained, the effect of the “NAM-B1 × weather conditions” interaction on productivity traits is lower than the contribution of other factors; and for the traits “the length of spike”, “the number of grains per spike”, and “the grain protein content”, it is statistically insignificant (see Supplementary Material 5).

It should be emphasized that NAM-B1 has a high degree of impact on the level of protein accumulation (70 %), which, along with a low contribution of weather conditions and the absence of genotype-environment interaction, indicates the effectiveness of functional allele introgression for the improvement of the quality of wheat grain in Belarus. A number of works by foreign scientists have shown a significant impact of the “NAM-B1 × environment” interaction on wheat productivity and grain quality traits. For example, the study by (Carter et al., 2012), established that differences in environmental conditions affect the expression of the NAM-B1 gene, which limits the use of functional allele introgression for increasing the level of protein accumulation in spring wheat grains in the regions with a short vegetation period. However, when studying the effects of NAM-B1 on the total protein content in grain and the main yielding components of common wheat in Argentina, for most of the traits studied (including thousand kernel weight and protein content), no significant interaction “NAM-B1 × environment” and “NAM-B1 × genotype” was shown, while the impact of “genotype” and “environment” factors was statistically significant (Tabbita et al., 2013).

## Conclusion

For the first time, full-length nucleotide NAM-A1 sequences of accessions T. kiharae, T. spelta k-1731, T. dicoccoides k-5199, and T. dicoccum k-45926 were identified and registered with the international molecular database GenBank. The studied accessions of related common wheat species had the NAMA1a haplotype, except for T. spelta k-1731 (NAM-A1c). Both NAM-A1d and NAM-A1a haplotypes are characteristic of T. aestivum varieties. Among the parental forms, a functional allele of the NAM-B1 gene was found only in the accessions of related species. Introgressive lines inherited, as a rule, the variants of NAM-1 genes of the original wheat variety. Out of 22 introgressive lines, the NAM-A1 haplotype of related species was identified in 6 lines, while a functional NAM-B1 allele was detected in 5. Line 13-3 T. dicoccoides × Festivalnaya (a/F ) and line 7 T. spelta k-1731 × Saratovskaya 29 (c/F ) had the NAM-А1 haplotype and the NAM-B1 allele of T. dicoccoides and T. spelta, correspondingly.

The genotyping results of introgressive lines of common wheat and parental forms by NAM-A1 and NAM-B1 genes were compared with the results of field trials in the conditions of Belarus and with the analysis data on protein content in grain of 2017–2021. The combination of NAM-A1/B1 genes had a significant effect on the formation of all the traits of wheat studied, while the grain weight per plant was statistically independent of the NAM-A1 haplotype, and the spike length and thousand kernel weight were statistically independent of the NAM-B1 allele. A joint contribution of these two genetic factors to the variability of economically valuable traits ranges from 8–10 % (grain weight per plant and thousand kernel weight) to 72 % (grain protein content).

For most of the traits studied, the proportion of variability determined by weather conditions was small (1.57–18.48 %). The closest correlation was established between the majority of traits studied and the HTC during the grain filling phase. The interaction “NAM-A1/B1 × weather conditions” determines 65–71 % of the variability of wheat grain productivity traits, while it is not significant for the grain protein content. The contribution of the “NAM-B1 × weather conditions” interaction to quantitative traits is lower than the contribution of other factors; and for the traits “spike length”, “the number of grains per spike” and “the grain protein content” it is statistically insignificant. It was found that the presence of a functional NAM-B1 allele provides for a high level of protein accumulation in grain, regardless of weather conditions, and at the same time, it does not lead to a significant decrease in thousand kernel weight.

Evaluation of the effect of six combinations of NAM-A1/B1 alleles on the level of manifestation of a number of economically valuable traits of wheat showed that high productivity of spike and plant but a low level of protein content in grain are characteristic of d/NF genotypes, while the highest protein concentration and low indicators by main productivity traits are characteristic of a/F. The optimal combination of the wheat traits studied was established for d/F genotypes. The results obtained prove the effectiveness of the introgression of a functional NAM-B1 allele of related species for increased nutritional value of common wheat.

## Conflict of interest

The authors declare no conflict of interest.
